# Establishment and validation of a simple and accurate qPCR detection method for *Haemophilus parasuis*

**DOI:** 10.1038/s41598-025-92803-1

**Published:** 2025-03-10

**Authors:** Hui Jin, Xingyu Xiao, Jingnan Wang, Zhihao Wang, Shuqin Wei, Changying Dong, Yongzhe Zhang, Chunyang Kang, Yajuan Sun

**Affiliations:** 1 Department of Neurology, China-Japan Union Hospital of Jilin University, 130033 Changchun, Jilin China; 2 College of Biological and Pharmaceutical Engineering, Jilin Agricultural Science and Technology University, 132101 Jilin, Jilin China; 3 College of Veterinary Medicine, Jilin Agricultural University, 130118 Changchun, Jilin China

**Keywords:** *Haemophilus parasuis*, *INFB*, Bearly detection, Clinical samples, Biochemistry, Molecular biology

## Abstract

As an infectious disease that poses a significant threat to the rapidly growing pig breeding industry, the detection of *Haemophilus parasuis* (HPS) is often compromised by various interfering substances present in the test sample during quantitative real-time PCR (qPCR). The rapid detection of HPS is important for the isolation of infectious pigs and their treatment. We designed and optimized a rapid qPCR test to detect the *INFB* gene of HPS in clinical and environmental samples on pig farms. The method was evaluated for its specificity, sensitivity, repeatability, anti-interference capability, and its ability to detect HPS in clinical samples. The results indicated that the method was specific for the detection of HPS when evaluated against pathogens and intestinal probiotics found in pig farms. By using a seven-fold dilution series of the recombinant plasmid DNA in triplicate, it was determined that the lowest limit of detection (LOD) for this method was less than 10 copies/µL. The results of inter-batch and intra-batch repeatability tests showed that the coefficient of variation (CV) was consistently below 1%. Furthermore, the impact of 14 endogenous and exogenous interfering substances on the Ct values detected by the HPS qPCR was found to be less than 5% when compared to the Ct values obtained in the absence of interfering substances. A total of 248 clinical samples were analyzed using the HPS qPCR, commercial kits, and corresponding national standards, yielding positive rates of 9.27%, 6.05%, and 9.27%, respectively. Notably, the positive and negative percent agreement between the detection method developed in this research and the national standard was 100%. These findings demonstrate that the established detection method is suitable for epidemiological research on HPS and for diagnosing clinical samples containing interfering substances, thereby providing essential technical support for the prevention and control of HPS.

**Supplementary Information:**

The online version contains supplementary material available at 10.1038/s41598-025-92803-1.

## Introduction

*Haemophilus parasuis* disease (HPSD), also known as polyfibrinous serositis and arthritis or Glaesser’s disease, is an infection in animals caused by *Haemophilus parasuis*(HPS) with pigs as the exclusive host^1,2^. In recent years, *H. parasuis* has been reclassified as *Glaesserella parasuis*^3^. Clinical signs of HPSD include fever, joint inflammation, breathing difficulties,and the presence of multiple serositis and arthritis^4,5^. This disease primarily affects pigs before and after weaning, as well as during the nursery stage, with an incidence rate ranging from 10–15%^6^. In cases of severe acute HPSD, the mortality rate in pigs can reach up to 50%, posing a significant threat to the health of piglets and young pigs^7^. The likelihood of the disease occurring increases when there are other infections such as reproductive respiratory syndrome, influenza, or enzootic pneumonia within the herd^8–10^. Due to the ubiquitous nature of HPS in the environment and its presence in both healthy and diseased pigs, HPS is a global issue that seriously impacts the swine industry^11–13^.

HPS is a gram-negative bacterium with variable morphology and over 15 serotypes, with serotypes 5, 4, and 13 being the most prevalent, comprising more than 70% of cases^14–16^. Approximately 20% of clinical strains remain untyped^17^. Variability in virulence and pathogenicity exists among different serotypes of HPS, with even strains of the same serotype demonstrating varying levels of pathogenicity and incidence rates^5,18^. The virulence of strains is closely linked to the virulence genes they harbor. Serotypes 1, 5, 10, 13, and 14 are highly virulent, while types 2, 4, and 15 are moderately virulent; other serotypes are considered non-virulent^5,18,19^. Extensive research has been conducted on the pathogenic mechanisms of HPS, immune response induction, and the development of vaccines. However, due to frequent co-infections with other pathogens and weak cross-protection between serotypes, vaccine efficacy is limited, resulting in widespread disease outbreaks that significantly impact the pig industry^2,20^. The rapid identification and physical isolation of sick pigs are the most effective methods for preventing and controlling the spread of HPSD. Consequently, there is an urgent need to develop rapid detection technologies for HPS.

Various methods have been developed for detecting HPS, including serological and molecular biology techniques^21–23^. Serological methods, including the complement fixation test, indirect hemagglutination test and enzyme-linked immunosorbent assay, exhibit limitations such as low analytical sensitivity and weak specificity during the early stages of pathogen infection^24–27^. In contrast, molecular biology methods like polymerase chain reaction (PCR), real-time fluorescence quantitative PCR (qPCR), loop-mediated isothermal amplification (LAMP), and nucleic acid molecular hybridization offer higher specificity and sensitivity. Among them, qPCR is the most widely used in virus detection. However, current qPCR methods for HPS detection lack specificity as they are based on the *16 S rDNA* sequence common to bacteria of the genus *Haemophilus*, leading to potential false positives^28^. Currently, existing HPS qPCR detection methods exhibit low sensitivity and may not be suitable for identifying low bacterial loads. Furthermore, the complexity of sample components significantly impacts the stability of the HPS qPCR reaction system. During the sample collection process, various exogenous and endogenous components are often introduced. These interfering substances, whether derived from the individual or the external environment, can interact with nucleic acids and impede the binding of primers to their templates. Additionally, these substances may directly inhibit the activity of enzymes involved in PCR reactions, thereby obstructing the normal progression of the reaction^29,30^.

Based on the above considerations, this study developed primers and probes based on the conserved region of the HPS *INFB* gene. By screening primers and optimizing the reaction system, a highly specific, sensitive, and low coefficient of variation HPS qPCR detection method was established. The HPS qPCR system described in this study is capable of rapidly detecting HPS in complex samples with low bacterial load, offering a new technical approach for the prevention and diagnosis.

## Materials and methods

### Samples and materials

The nucleic acids employed to assess the specificity of our method for detecting HPS in clinical samples were extracted from a range of viral vaccines and bacteria in this study. These included the porcine mycoplasma pneumonia inactivated vaccine, porcine atrophic rhinitis inactivated vaccine (Bordetella JB5 strain), porcine parvovirus inactivated vaccine (WH-1 strain), Porcine circovirus disease type 2 inactivated vaccine (SH strain), swine fever, swine erysipelas, porcine pasteurellosis multocida triple live vaccine, porcine pseudorabies live vaccine (HB-98 strain), *Enterococcus faecalis*, and *Clostridium butyricum* which were purchased from vaccine companies and probiotic companies. Detail information can be found in Supplement Table S1. Additionally, *Escherichia coli*, *Bacillus subtilis*, and *Salmonella* were isolated and stored in our laboratory. The analytical grade ethanol and isopropanol (purity ≥ 99.7%) utilized in this experiment were acquired from Samson Chemical Technology (Shanghai) Limited Company.

Samples were collected from pigs exhibiting clinical symptoms of HPS, as well as from their living environments in pig raising enterprises located in Jilin Province. These samples were obtained through random sampling. The total number of clinical samples analyzed was 248, comprising 56 whole blood samples, 48 serum samples, 32 swab samples from the oral cavity, nose, and anus, and 74 tissue samples (lung, spleen, liver, kidney, tonsil, and lymph node). Additionally, there were 26 saliva samples and 12 environmental samples, all of which were stored at 4℃ for future use.

### Nucleic acid extraction

Nucleic acids were extracted from vaccines, constructed plasmids, and clinical samples using the magnetic bead bacterial total nucleic acid extraction kit and the magnetic bead viral total nucleic acid extraction kit, both purchased from Sairuisi Biotechnology Co., Ltd. (Jilin, China). For blood and faeces, the volume of the lysate or duration of water bath incubation was amended according to kit instructions for nucleic acid extraction for these sample types. The extracted nucleic acids were then stored at −20 °C for future use.

### Primer and probe design

Primer and probe design utilized the published GenBank numbers of 15 HPS serotypes randomly obtained from NCBI. The registration numbers and corresponding serotypes of HPS are shown in Table 1. Utilize Vector NTI Advance (11.5.1) to align the downloaded gene sequences and design primers (Figure S1). The designed primers and probes were then evaluated using PrimeSelect software to assess physical parameters, including annealing temperature, hairpin structure, primer-dimer formation, and free energy. The specificity of the primers and probes was verified using the BLAST tool provided by NCBI (http://www.ncbi.nlm.nih.gov/blast). Subsequently, primers with similar annealing temperatures, high specificity, low free energy, and minimal formation of primer dimers and hairpin structures were selected for synthesis. The cytoskeletal housekeeping gene, *β-actin*, is expressed in pig cells and was used as an internal control to ensure nucleic acids were recovered from porcine samples. The synthesis of primers and probes was conducted by Sangon (Shanghai, China). The sequences of the primers and probes are detailed in Table 2.

**Table 1:** Accession numbers and corresponding serotypes of HPS.

Accession numbers	Serotypes
WCKA00000000	1
APCB01000037	2
NZ_APBU00000000	3
CP040243	4
CP021644	5
CP069308	6
CP049088	7
WCJR00000000	8
NZ_APBZ00000000	9
WIUS00000000	10
NZ_APBY00000000	11
CP005384	12
WCKJ00000000	13
WCJS00000000	14
NZ_APBX00000000	15

**Table 2:** Primer and probe sequences for the detection of HPS.

Primer/Probe	Sequences(5’- 3’)	Product length(bp)
HPS-F1	GCTTTCGATAATGCGACGTGCT	161 bp
HPS-R1	TGCCGTTGAAAGCTCGTGTAAAGA	
HPS-P1	VIC- TACCACCTACGCCAGAGCCAACC -TAMRA	
HPS-F2	GCCTGAAAGTCCTAATACTTCCACA	286 bp
HPS-R2	GGCTTCAAGTAAGTCGTCAATCCCCATT	
HPS-P2	VIC- GCACTTAATTCTAATACTTCCGATTGA -MGB	
*β-actin*-F	TGCACAGTAGGTCTGACGTG	88 bp^31^
*β-actin*-R	CCAGACTGGGGACATGCAG	
*β-actin*-P	FAM-TCCCCAGCACACTTAGCCGTGTT-BHQ1	

HPS-F1/R1/P1 refers to the initial set of primer probes designed for HPS detection, while HPS-F2/R2/P2 denotes the second set of primer probes for the same purpose. The *β-actin*-F/R/P primer probe is specifically designed for the porcine housekeeping gene *β-actin.*

### Procedures and systems of amplification reaction

In this study, three PCR methods were employed: conventional PCR, SYBR Green I qPCR, and TaqMan qPCR. The primary function of conventional PCR is to amplify the target fragment, which is subsequently purified using gel electrophoresis and utilized for plasmid construction. The primers utilized in this process are HPS-F1/R1, as listed in Table 1. Both SYBR Green I qPCR and TaqMan qPCR were implemented to evaluate the amplification efficacy of the two primer pairs, HPS-F1/R1 and HPS-F2/R2, also detailed in Table 2. The amplification reaction systems, amplification conditions, and the concentrations of primers and probes used for the three PCR methods are outlined below.

The conventional PCR system used in this study involved combining 2 x PCR Master Mix (Sangon, Shanghai, China) 10 µL, 0.5 µL each of forward and reverse primers (10 µM), 2 µL template, and ddH_2_O to make up to a total volume of 20 µL. The PCR amplification program included an initial pre-denaturation step at 95℃ for 5 min, followed by 40 cycles of denaturation at 95℃ for 30 s, annealing at 55℃ for 30 s, extension at 72℃ for 45 s, and a final extension at 72℃ for 10 min. This programme is used for plasmid construction.

The SYBR Green I qPCR reaction system consisted of 2 x RealStar Fast dye qPCR premix (UNG, High ROX) (Kangrun, Beijing, China) 10 µL, 0.5 µL each of the forward and reverse primers (10 µM), DNA template 1 µL, and ddH_2_O to a final volume of 20 µL. The reaction protocol included UNG enzyme treatment at 50℃ for 5 min, followed by pre-denaturation at 95℃ for 2 min, and 40 cycles of reaction (denaturation at 95℃ for 15 s, annealing at 60℃ for 30 s). The melting curve analysis was performed automatically using a StepOnePlus real-time fluorescence quantitative PCR instrument (Thermo Fisher, Waltham, MA, USA). Thresholds are set automatically by the machine.

TaqMan qPCR was performed using 2xTaq Pro U^+^ Multiple qPCR Mix (Nazyme, Nanjing, China), with 10 µL total volume. The reaction mixture included 0.2 µL each of forward and reverse primers (10 µM), 0.1 µL probe (10 µM), 0.4 µL of 50x ROX Reference Dye, 2 µL of DNA template, and ddH_2_O to a final volume of 20 µL. The reaction program consisted of a contamination digestion step at 37℃ for 2 min, followed by pre-denaturation at 95℃ for 30 s, and 40 cycles of reaction (denaturation at 95℃ for 10 s, annealing at 60℃ for 30 s). The amplification products generated during repeated qPCR amplification are a primary source of aerosol contamination of nucleic acid amplicons. This aerosol contamination is a significant contributor to false positive detection results. To mitigate the risk of false positive HPS results caused by these nucleic acid amplicon aerosols, a UNG enzyme anti-contamination system was incorporated into the TaqMan qPCR amplification system.

### Recombinant plasmid Preparation

The genomic DNA of the HPS strain was amplified using a selected primer set (HPS-F1/R1) through PCR. Following PCR amplification, the product underwent 2% agarose gel electrophoresis to isolate the target band, which was then ligated with the pMD19-T plasmid vector (Takara, Beijing, China) and introduced into *E. coli* DH5α competent cells (Sangon, Shanghai, China). Enrichment culture and screening of positive clones were carried out. The success of plasmid construction was determined by PCR amplification and sequencing results. The successfully constructed positive plasmid was named pMD19-*INFB* and stored for future use. Nucleic acids from the positive plasmid pMD19-*INFB* were extracted according to the method described in Sect. 2.2, and subsequently, the gene copy number corresponding to 1 ng of DNA was calculated using the following formula.

copies/mL = 6.02 × 10^23^(copies/mol) × (concentration g/mL) / (MW g/mol)^32,33^.

### Primer and probe selection

Using the DNA from the recombinant plasmid pMD19-*INFB* as the amplification template, both the SYBR Green I staining method and the TaqMan probe method were employed to screen the primers and probes listed in Table 2. The absence of primer-dimers in the melting curve and low Ct values were utilized as criteria for screening HPS primer probes.

### Optimization of HPS qPCR system

In order to determine the optimal qPCR reaction conditions, the primer, probe concentrations, and annealing temperature were optimized using plasmid DNA templates at concentrations of 3.16 × 10^3^ copies/µL, 3.16 × 10^4^ copies/µL, and 3.16 × 10^5^ copies/µL. The primer volume range tested were 50 nM, 100 nM, and 150 nM (with the probe concentration held constant at 50 nM). After identifying the optimal primer concentration, probe volume optimization was conducted with levels of 50 nM, 100 nM, and 150 nM. Subsequently, annealing temperature optimization was carried out with temperatures of 58 °C, 60 °C, and 62 °C. Each experiment was repeated three times.

### Specificity test

In this study, 11 pathogens commonly found in swine farms were selected for specificity testing. These pathogens included *Streptococcus suis* (SS), *Escherichia coli* (ED), *Erysipelas reinhardtii* (Ery), *Actinobacillus pleuropneumoniae* (APP), *Mycoplasma pneumoniae* (Mp), *Salmonella porcine*, *Pasteurella* porcine (PRDC), infectious rhinitis of pig (AR), *pseudorabies virus* (PRV), *porcine papillomavirus* (PPV), and *porcine circovirus 2* (PCV2). Additionally, nucleic acids of three porcine companion probiotics (*Clostridium butyricum*,* Enterococcus faecalis*, and *Bacillus subtilis*) were also used for specificity validation of the HPS TaqMan qPCR method. The specificity experiments involved using a standard plasmid with 3.16 × 10^6^ copies/µL as a positive control and ddH_2_O as a negative control. The amplification process followed optimized reaction conditions and was repeated three times for accuracy.

### Establishment of standard curves

The recombinant plasmid pMD19-*INFB* was utilized as a qPCR template after being diluted 10-fold to a concentration ranging from 10^2^ copies/µL to 10^6^ copies/µL. The optimized reaction system and conditions were employed for a single qPCR amplification, and a standard curve was generated by plotting the logarithm of the initial copy number concentration of the plasmid standard against the Ct value.

### Lowest limit of detection (LOD)

To determine the lowest detection limit of the established HPS qPCR, we diluted the plasmid DNA to a concentration range of 3.16 × 10^7^ copies/µL to 3.16 × 10^0^, and utilized an optimized final qPCR reaction system for amplification. The minimum plasmid concentration that successfully generated an amplification curve was defined as the minimum detection limit. Each concentration was tested in triplicate to ensure the accuracy of the experiment. Additionally, ddH_2_O was utilized as a no template control during the amplification process to validate the reliability of the experimental procedure. Subsequently, the sensitivity results of the qPCR assay guided the selection of an appropriate concentration range for conventional PCR amplification. In addition, the performance of the HPS qPCR reaction system was further assessed by comparing it with commercially available kits(Weiboxin, Guangzhou, China) using genomic DNA extracted from HPS vaccines, which were subjected to a tenfold gradient dilution as a template.

### Precision test

To assess the repeatability of the HPS qPCR assay system, qPCR amplification was conducted with standard plasmids at three different concentrations (3.16 × 10^3^ copies/µL, 3.16 × 10^4^ copies/µL, and 3.16 × 10^5^ copies/µL) to evaluate the intra- and inter-batch precision of the experiments. Intra-batch precision was assessed by using the same batch of prepared qPCR reaction systems and performing three replicate amplifications for each template concentration. Inter-batch precision was evaluated by preparing 5 batches of qPCR reaction systems over 5 working days and conducting 3 amplifications for each template concentration. The coefficient of variation (CV) was calculated as the standard deviation (SD) divided by the mean, multiplied by 100%. If the coefficient of variation (CV) value is lower than 5%, the established HPS qPCR detection system is deemed to exhibit good repeatability.

### Interference test

The HPS assay samples are often collected in complex environments, where they may contain various interfering substances that can greatly impact the sensitivity and reliability of the assay results. To assess the tolerance of the HPS qPCR assay system to potential interfering substances, an experimental protocol was developed based on the specific clinical samples and potential interferers present. In this protocol, 100 µL of a medium positive plasmid standard (3.16 × 10^5^ copies/µL) was mixed with either 8 endogenous interfering substances or 6 exogenous interfering substances as the amplification template, and an optimized qPCR system was utilized for amplification. Endogenous interfering substances refer to physiological components present in the test sample that can interfere with substance analysis, including mucins, latex, blood, heart, liver, spleen, lungs, and intestine. In contrast, exogenous interfering substances are those originating from outside the body that can also affect substance analysis, including Reagent 1–4, feces and feeds. Additionally, a positive control consisting of 100 µL of medium-positive plasmid standard mixed with 100 µL of ddH_2_O was included, along with a negative control of 200 µL of ddH_2_O to assess the impact of potential interferers on the assay. Each set of experiments was conducted in triplicate, and the final results were reported as the mean Ct value ± standard deviation (SD). An increase of 5% in the Ct value of the interference group compared to the positive control is classified as slight inhibition, whereas an increase of 10% is deemed significant inhibition.

### Testing of clinical samples

DNA was extracted from the clinical samples described in Sect. 2.1, and HPS was detected using the method established in this study, along with the purchased HPS nucleic acid detection kit (Wei Boxin, Guangzhou, China). The national standard for HPS detection, as outlined in SN/T4230-2015 “Technical Specifications for Quarantine of *Haemophilus parasuis*,” served as the gold standard for evaluating the detection efficacy of the two methods. To ensure the accuracy of the test results, the positive control provided in the purchased kit was utilized to confirm successful amplification. Additionally, the entire extraction process was monitored using the porcine housekeeping gene *β-actin*. The amplification process was controlled with water as a no template control, and an open tube control was employed to evaluate the extraction environment. This comparison was conducted using three indicators: positive percent agreement, negative percent agreement and Cohen’s Kappa. The calculation method for positive and negative percent agreement involves determining the number of samples classified as positive or negative by both the HPS qPCR (commercial kit) and the national standards. This value is then divided by the number of samples classified as positive or negative by the national standards alone, and the result is multiplied by 100%.

## Results

### Results of primer probe screening

**Fig. 1:**
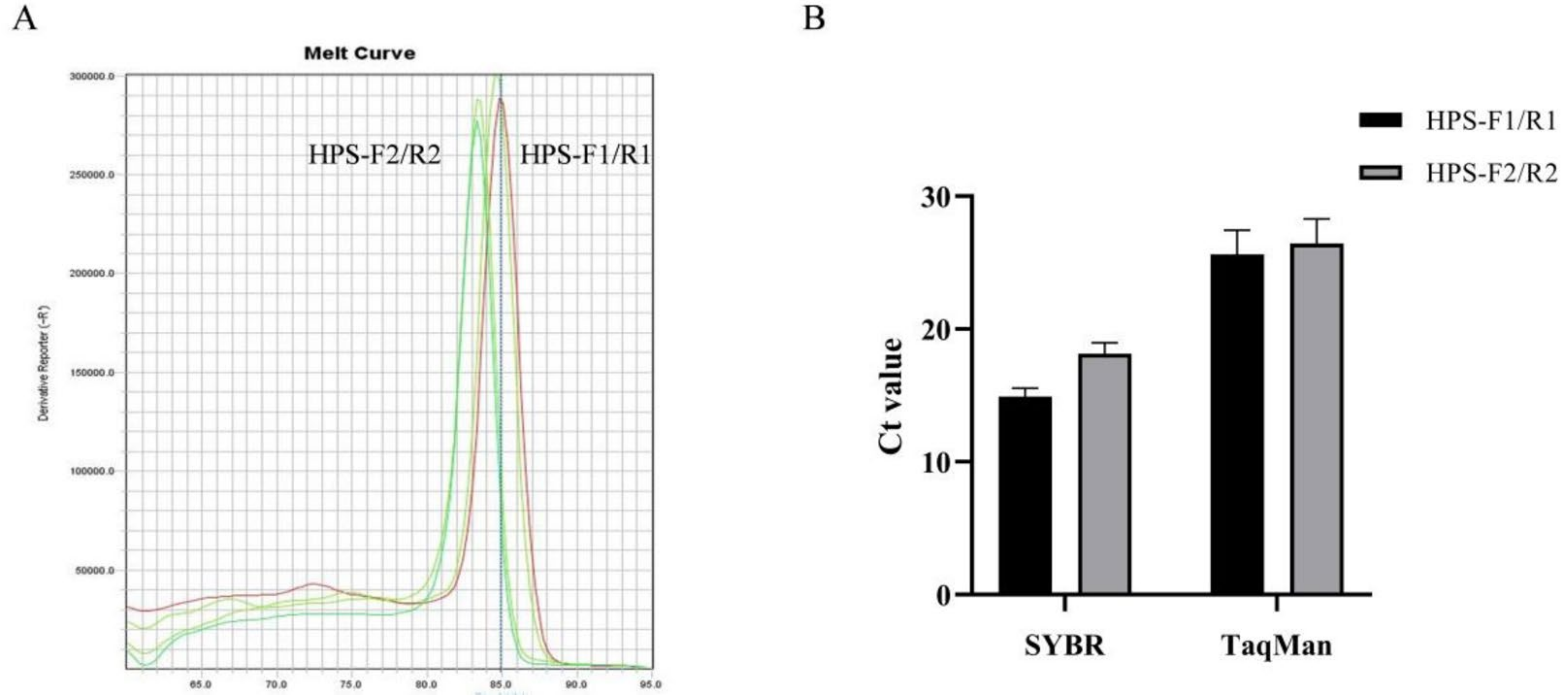
(**A**) Melting curves of HPS-F1/R1 and HPS-F2/R2; (**B**) Comparison of amplification effects of HPS-F1/R1 and HPS-F2/R2.

To improve amplification, primers were initially screened using both SYBR Green I and TaqMan probe methods. Melting curves indicated that HPS-F1/R1 and HPS-F2/R2 were devoid of Primer dimers and non-specific amplification (Fig. 1A). Further screening based on Ct value revealed that HPS-F1/R1 showed superior amplification results with both SYBR Green I and TaqMan probe methods (Fig. 1B). Consequently, HPS-F1/R1 and its corresponding probe were chosen for subsequent experiments.

### Identification of plasmid pMD19-*INFB*

**Fig. 2:**
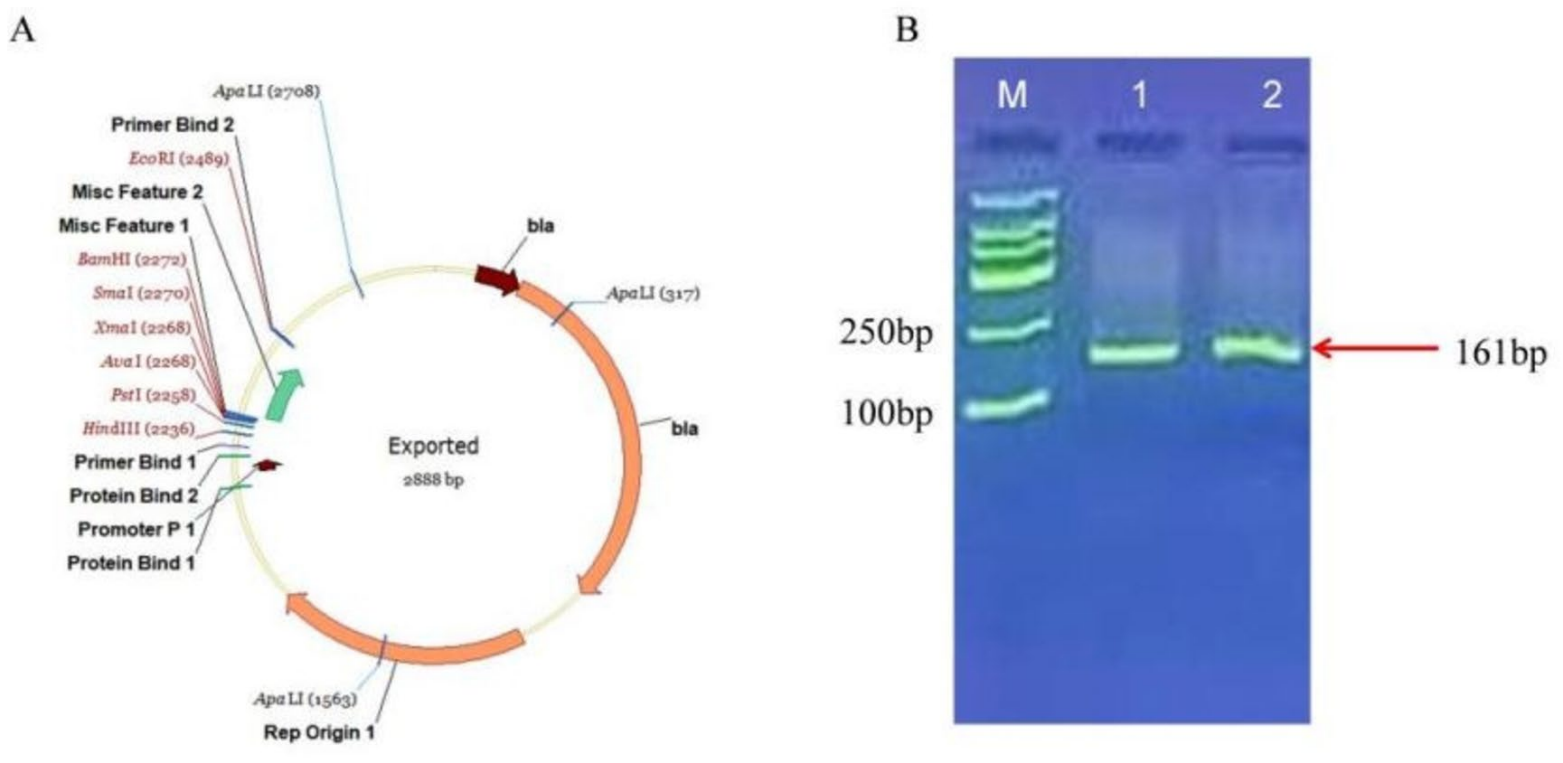
(**A**) pMD19-INFB plasmid construction; (**B**) Gel electrophoresis results of PCR amplification products; M is Marker with molecular weight 2000, 1 and 2 are amplification products.

Figure 2A illustrates the constructed recombinant plasmid. The PCR amplification product of the plasmid DNA ranged from 100 to 250 bp, confirming the successful construction of the plasmid (Fig. 2B). Additionally, sequencing results further validated the successful construction of the plasmid. Consequently, this constructed plasmid is suitable for use in subsequent experiments. Furthermore, based on the copy number calculation formula, 1 ng of plasmid DNA corresponds to approximately 3.16 × 10^9^ copies/µL.

### Optimization of HPS qPCR reaction system

To identify the most effective reaction system for HPS qPCR detection, this study systematically optimized key conditions using three different concentrations of recombinant plasmid DNA. The results are detailed in Table 3. It was found that with a primer concentration of 150 nM, probe concentration of 100 nM, and an annealing temperature of 60 °C, the reaction system demonstrated the most efficient amplification. This optimized qPCR reaction system can now be utilized for future experiment.

**Table 3:** Optimization of primer and probe concentration and annealing temperature.

Factor	Parameters	Plasmid concentration(copies/µL)		
		3.16 × 10^5^	3.16 × 10^4^	3.16 × 10^3^
Primer concentration (nM)	50	22.79 ± 0.16	27.58 ± 0.54	30.48 ± 0.01
	100	22.67 ± 0.25	27.25 ± 0.08	30.38 ± 0.07
	150	22.43 ± 0.03	26.94 ± 0.06	29.96 ± 0.03
Primer concentration(nM)	50	23.41 ± 0.09	26.47 ± 0.20	30.34 ± 0.08
	100	22.61 ± 0.16	25.91 ± 0.05	29.66 ± 0.05
	150	22.67 ± 0.02	25.90 ± 0.10	29.49 ± 0.05
Annealing temperature (℃)	58	24.20 ± 0.03	27.70 ± 0.11	31.03 ± 0.11
	60	24.08 ± 0.01	27.64 ± 0.09	30.91 ± 0.11
	62	24.17 ± 0.01	27.64 ± 0.17	31.08 ± 0.01

### Specificity of HPS qPCR

**Fig. 3:**
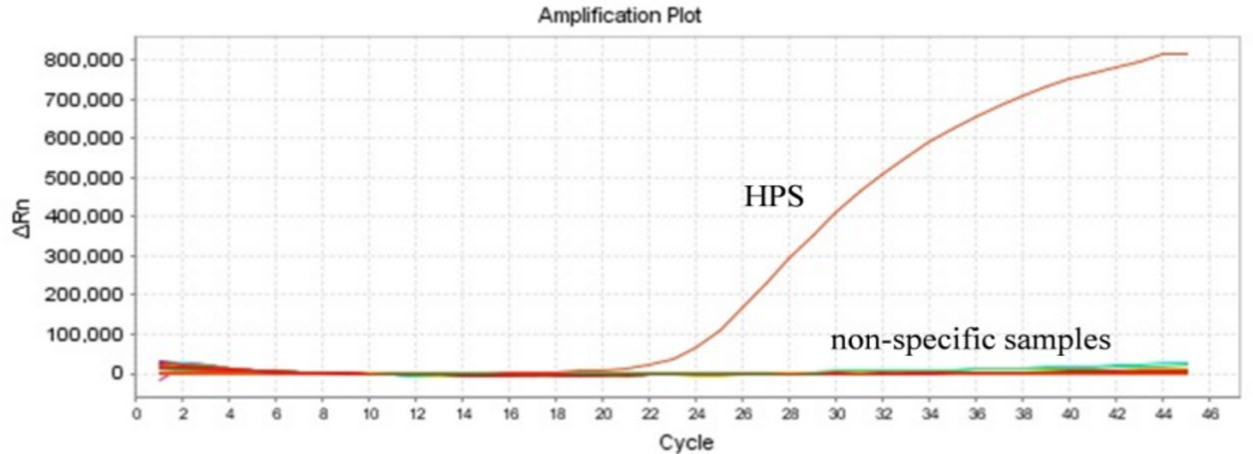
HPS qPCR specificity assay.

In order to validate the specificity of the HPS qPCR method, nucleic acids were extracted from 11 common pathogens found in pig farms and 3 types of pig-associated probiotics. Subsequently, qPCR amplification was performed. As shown in Fig. 3, the detection method established in this study demonstrated specific amplification solely for HPS nucleic acid samples, without showing specific amplification for 11 common pathogens and 3 pig-related probiotics. Consequently, the qPCR detection method designed in this study exhibited strong specificity in detecting common pig pathogens.

### Establishment of HPS qPCR standard curve

**Fig. 4:**
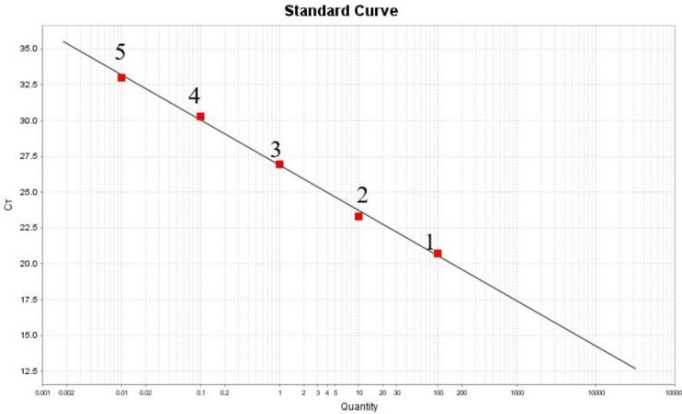
Standard curve for TaqMan qPCR of pMD19-*INFB*.

The standard curve of HPS positive plasmid is illustrated in Fig. 4, showing a linear relationship between plasmid copy number and Ct value with the equation y = −3.415x + 19.669. The correlation coefficient (R^2^) is 0.997, and the amplification efficiency is 96.26%. These results indicate that the qPCR assay for HPS developed in this study exhibits a strong linear relationship within the range of 3.16 × 10^2^ − 3.16 × 10^6^ copies/µL.

**Fig. 5:**
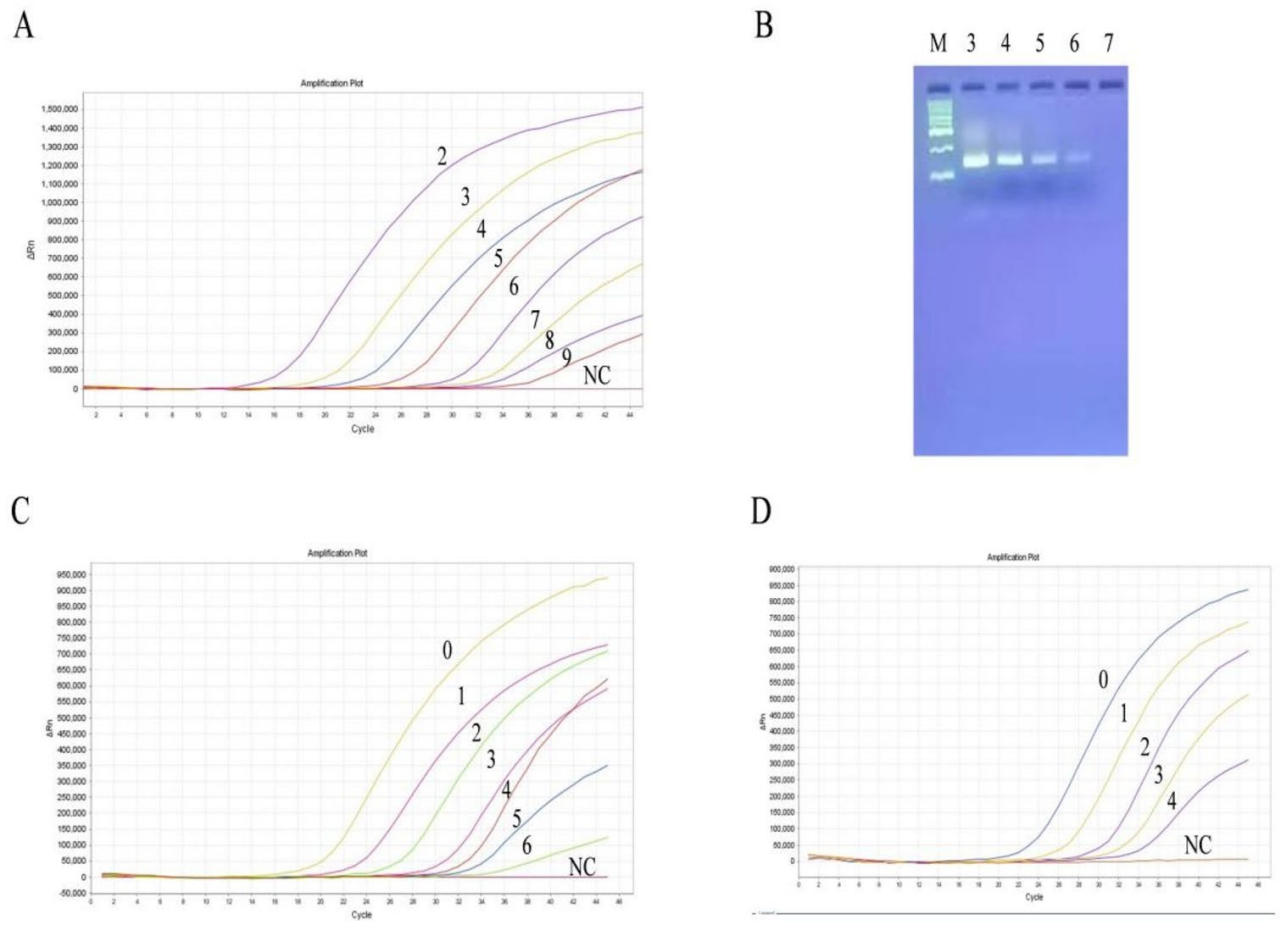
(**A**) Sensitivity of qPCR HPS detection; (**B**) Sensitivity of normal PCR detection; (**C**) Sensitivity of HPS qPCR established in this study for detection of viral nucleic acids; (**D**) Sensitivity of commercial kits for detection of viral nucleic acids. NC: Negative control; M: Marker with molecular weight 2000; All numbers in A-D indicate the number of dilutions.

### Sensitivity assay for HPS qPCR

The qPCR amplification of the diluted DNA from positive plasmid demonstrated that the HPS detection system developed in this study could detect as low as 3.16 copies/µL (Fig. 5A). In contrast, conventional PCR only displayed weak bands in the electrophoresis channel when the plasmid concentration reached 3.16 × 10^3^ copies/µL (Fig. 5B). This indicates that the system developed in this study is 1000 times more sensitive than conventional PCR. Furthermore, the system was able to specifically detect vaccine nucleic acid at a 10^6^-fold dilution (Fig. 5C), whereas the competitor reagent could only detect it at a 10^4^-fold dilution (Fig. 5D). Overall, the method established in this study shows a analytical sensitivity that is 100 times higher than that of commercial kits. It is important to note that while high analytical sensitivity contributes to improved diagnostic sensitivity, it does not necessarily equate to diagnostic sensitivity itself.

### Precision experiment for HPS qPCR

Three different concentrations of pMD19-*INFB* DNA were chosen for testing, and the optimized systems were utilized for both intra-batch and inter-batch repeated experiments. As shown in Table 4, The coefficient of variation for each group in repeated experiments was less than 1%, indicating that the HPS qPCR method developed in this study demonstrates excellent repeatability.

**Table 4:** Reproducibility of the Real-Time qPCR assay (*n* = 3).

Concentrations of standard plasmid	Intra-batch variance		Inter-batch variance	
	Mean ± SD	CV (%)	Mean ± SD	CV (%)
3.16 × 10^6^ copies/µl	20.11 ± 0.04	0.20	20.96 ± 0.16	0.76
3.16 × 10^5^ copies/µl	23.44 ± 0.08	0.34	24.40 ± 0.15	0.61
3.16 × 10^4^ copies/µl	27.05 ± 0.09	0.33	27.74 ± 0.21	0.76

### Results of HPS qPCR interference experiments

The stability of the established HPS qPCR detection system was confirmed by testing with various types of endogenous and exogenous interfering substances. Table 5 indicates that the presence of feces, mucin, and fecal matter in the test samples slightly inhibited the detection efficacy of HPS. However, no interfering substances resulted in severe inhibitory effects. These findings demonstrate that the HPS qPCR detection system developed in this study exhibits strong resistance to a range of endogenous and exogenous interfering substances.

**Table 5:** Interference experiment results.

Number	interfering materials	Mean of Ct
1	Reagent 1^a^	21.49
2	Reagent 2^b^	20.88
3	Reagent 3^c^	20.98
4	Reagent 4^d^	21.32
5	Mucins	22.55
6	Feeds	21.34
7	Faeces	23.16
8	Latex	22.92
9	Blood	21.88
10	Heart	21.43
11	Liver	21.44
12	Spleen	21.56
13	Lungs	20.98
14	Intestine	21.24
15	positive controls	21.19
16	negative controls	N/A

a: Equivalent mixture of ribavirin and amantadine; b: Equivalent mixtures of amoxicillin, doxycycline and tamsulosin; c: Equivalent mixture of ceftiofur, florfenicol and tilmicosin; d: Equivalent mixture of dexamethasone and gentamicin. where 1–7 are exogenous interfering substances and 8–16 are endogenous endogenous interfering substances.

### Clinical sample testing

**Fig. 6:**
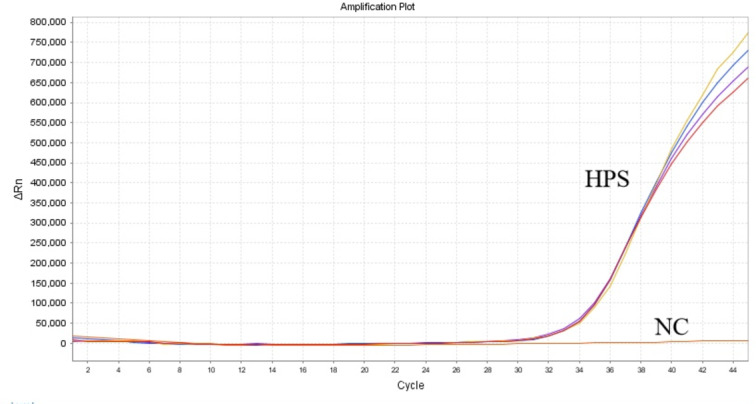
HPS sample retest results.

In this study, 248 samples were tested using both the method developed here and commercially available kits. As shown in Table 6, Using national standards as the benchmark, the probability of a sample being identified as positive by the control kit was 6.05% (95% CI 4.08%−8.02%), while the probability of a sample being identified as positive by our method was 9.27% (95% CI 7.07%−11.47%). the positive and negative percent agreement indicates that the detection method developed in this study performs comparably to the national standard. Samples that tested positive with the experimental system but negative with the competitor’s kit were retested to confirm the positive results (Fig. 6). Furthermore, with the national standard as the reference and Cohen’s Kappa as the evaluation index, the κ value of the control kit was 0.776, while the κ value of our study was 1. These results clearly demonstrate that the qPCR detection system developed in this study has a higher detection rate compared to the competitor kits.

**Table 6:** Detection effects of HPS by different methods.

Clinical samples (248)	Control kits	HPS qPCR	National standards.	
Positive sample	15	23		23
Negative sample.	233	225		225
Positive rate	6.05%	9.27%		9.27%
Positive percent agreement	65.21%	100%		**/**
Negative percent agreement	100%	100%		**/**
Cohen’s Kappa (κ)	0.776	1		**/**

“/ ”: Not applicable.

## Discussion

HPS is commonly found in the environment. This slow-growing bacterium has specific nutritional requirements and is highly sensitive to fluctuations in its surrounding environment^34,35^. Consequently, any alterations in culture conditions during the collection and transportation of HPS samples to the laboratory may lead to a reduction in HPS survival. This difficulty further limits the use of bacterial isolation and culture as the ‘gold standard’ for diagnosing HPS^36–38^. Pigs are frequently co-infected with HPS and other bacteria and viruses. In instances of co-infection, the mortality rate among pigs affected by HPS is significantly elevated^39,40^. Furthermore, the weak cross-protection observed between HPS serotypes considerably diminishes the efficacy of vaccines^12,41^, thereby increasing the likelihood of large-scale epidemic outbreaks. Consequently, regardless of vaccination status, timely and regular detection of HPS is essential, as it can effectively reduce the economic losses associated with pigs infected by HPS.

Currently, the mainstream methods used for HPS detection are categorized into serological and molecular biology techniques. Due to the high cost associated with individual tests and the challenges in detecting early infections using serological diagnostic methods, molecular biology approaches are frequently employed in practical applications. Among these, qPCR technology offers advantages in terms of a mature technical system and high detection sensitivity when compared to conventional PCR, loop-mediated isothermal amplification, and nucleic acid molecular hybridization. In conventional PCR, the *16 S rRNA*gene is commonly utilized as a target for primer design, serving as a taxonomic and phylogenetic marker^35,42^. However, in qPCR diagnostics, no significantly distinct regions within the *16 S rRNA* gene have been identified between the primer sequences of HPS and other species, making it difficult to differentiate *H. parasuis*from closely related species^43^. Research conducted by C. Turni et al. (2010). has indicated that *IFNB* serves as a reliable surrogate target for *16 S rRNA*, effectively distinguishing *H. parasuis*from all other closely related species^35^. Currently, numerous qPCR diagnostic methods targeting the *IFNB*gene for HPS have been developed^44–46^. This study has also designed detection primers and probes based on the conserved region of the housekeeping *INFB* gene across 15 serotypes of HPS to facilitate HPS detection.

To prove the resistance of the established HPS qPCR to these interfering substances, we mixed several potential interferents with the amplified nucleic acid samples for experimentation, a consideration that was absent in previous HPS qPCR studies. The results indicate that the qPCR detection system developed in this study exhibits strong anti-interference capability. Among all tested substances, feces, mucin (from semen), and milk were found to inhibit the detection efficacy of HPS. This may be attributed to the high concentrations of these interfering substances, which contain impurities and nucleic acids that are challenging to separate. Furthermore, aerosol contamination is a primary contributor to false positive test results^47,48^. In this study, we incorporated a UNG enzyme contamination prevention system into the qPCR amplification framework, effectively eliminating false positives caused by nucleic acid amplicon aerosols.

This study has several limitations. Although we verified the specificity of the primer probes using bioinformatics methods during the primer design process, we were unable to obtain species for comparison with HPS. Consequently, during the specificity detection phase, only common pig pathogens were utilized for specific detection. Furthermore, in the HPS qPCR process, HPS strains were not isolated from the samples and cultured for identification, which constitutes another limitation of this study. However, subsequent identification of real samples may partially address this limitation.

## Conclusion

The study utilized the *INFB* gene of HPS for primer probe design and optimized the primer concentration, probe concentration, and annealing temperature in the reaction system through primer screening. The optimal reaction conditions for HPS qPCR were determined as follows: using F1/R1 as the primer, a primer concentration of 0.3 µL, a probe concentration of 0.2 µL, and an annealing temperature of 60℃. The established detection system specifically amplified HPS among common pig pathogens, achieving a detection sensitivity of less than 10 copies/µL. The coefficient of variation (CV) values for both intra-batch and inter-batch repeatability experiments were below 1%. Additionally, the system demonstrated good resistance to 8 endogenous and 6 exogenous interfering substances. Ultimately, the positive detection rate of HPS in real sample testing surpassed that of competing kits.

## Electronic supplementary material

Below is the link to the electronic supplementary material.

Supplementary Material 1

## Data Availability

The datasets used and analysed during the current study available from the corresponding author on reasonable request.
