# Supplementary material for: Establishment and validation of a simple and accurate qPCR detection method for Haemophilus parasuis
Source: Sci Rep. 2025 Mar 10;15:8264. doi: 10.1038/s41598-025-92803-1 (PMC11893755; doi:10.1038/s41598-025-92803-1)
Supplement: Supplementary file 1 — Supplementary Material 1 [file 41598_2025_92803_MOESM1_ESM.docx]

**Supplement Table S1 Sources of disease materials**

| Name | Product Specification | Source |
| --- | --- | --- |
| Streptococcus suis propolis inactivated vaccine | 20mL/bottle | Shandong Huahong Biological Engineering Co. |
| Tetravalent inactivated propolis vaccine for haemophilus parvovirus disease | 20mL/bottle |  |
| Trivalent inactivated porcine pleuropneumonia vaccine | 20mL/bottle | Wuhan Keqian Biological Co. |
| Mycoplasma pneumoniae inactivated vaccine | 20mL/bottle |  |
| porcine atrophic rhinitis inactivated vaccine (Bordetella JB5 strain) | 20mL/bottle |  |
| porcine parvovirus inactivated vaccine (WH-1 strain)） | 20mL/bottle |  |
| Porcine circovirus disease type 2 inactivated vaccine (SH strain) | 20mL/bottle | Pulaike Bioengineering Co. |
| swine fever, swine erysipelas, porcine pasteurellosis multocida triple live vaccine | 10 heads/bottle | Chongqing Yuanquan Animal Protection Technology Co. |
| porcine pseudorabies live vaccine (HB-98 strain) | 10 heads/bottle | Zhongmu Industry Co. |
| *Enterococcus faecalis* | 10g/bottle | Nongfukang Biotechnology Co. |
| Clostridium butyricum | 10g/bottle |  |


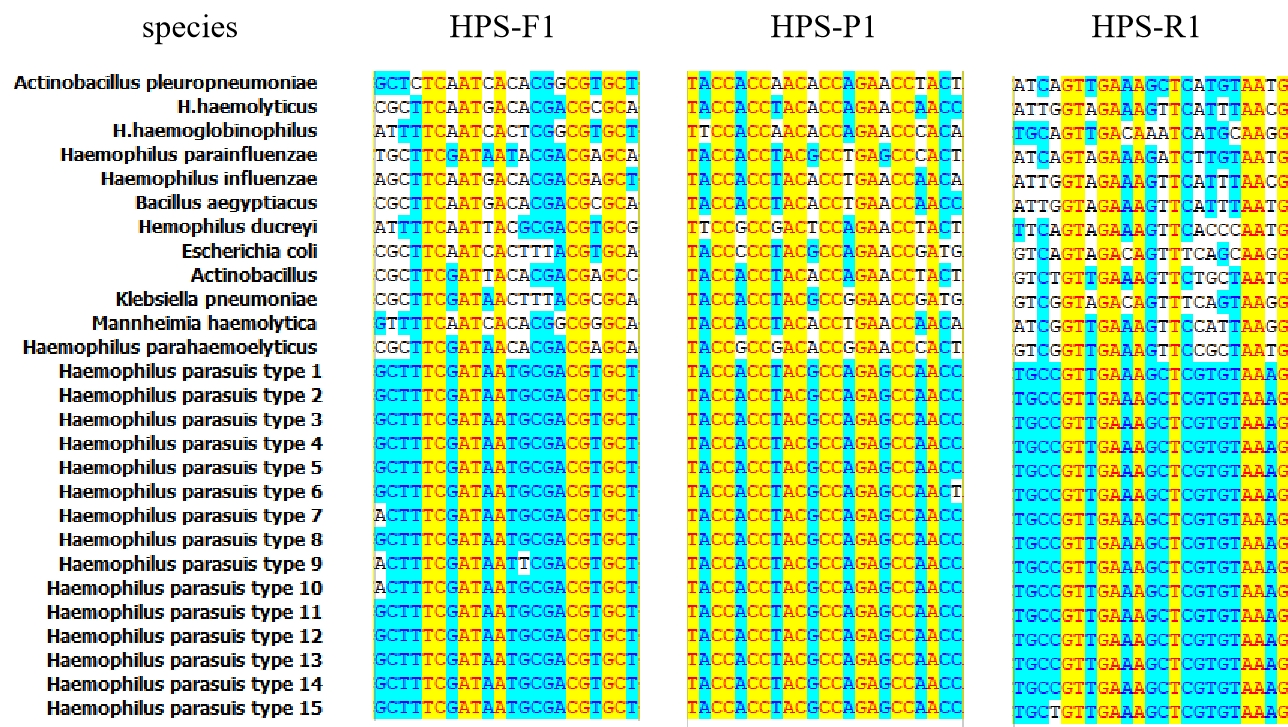


**Figure S1 Sequence alignment results of HPS**


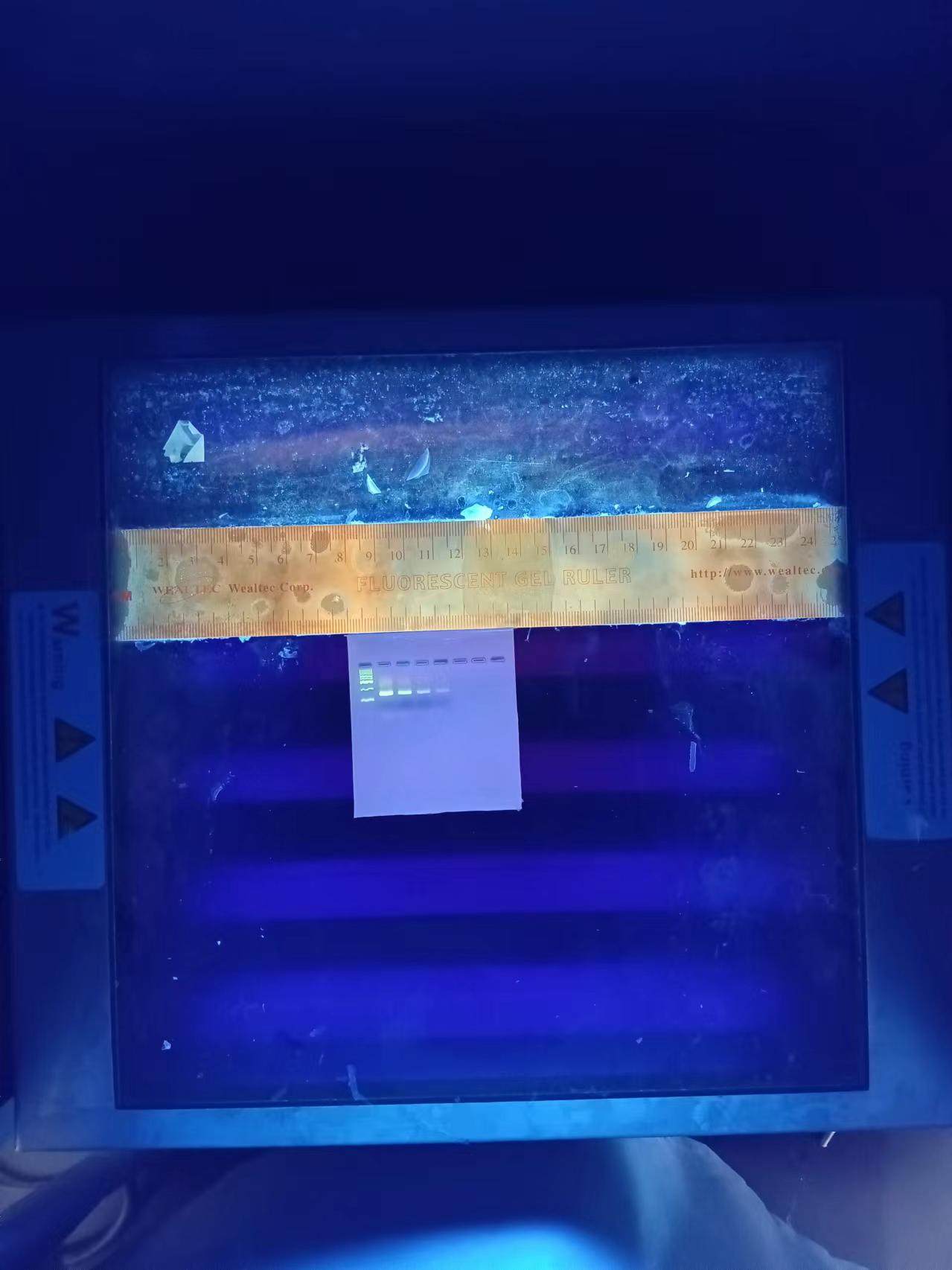

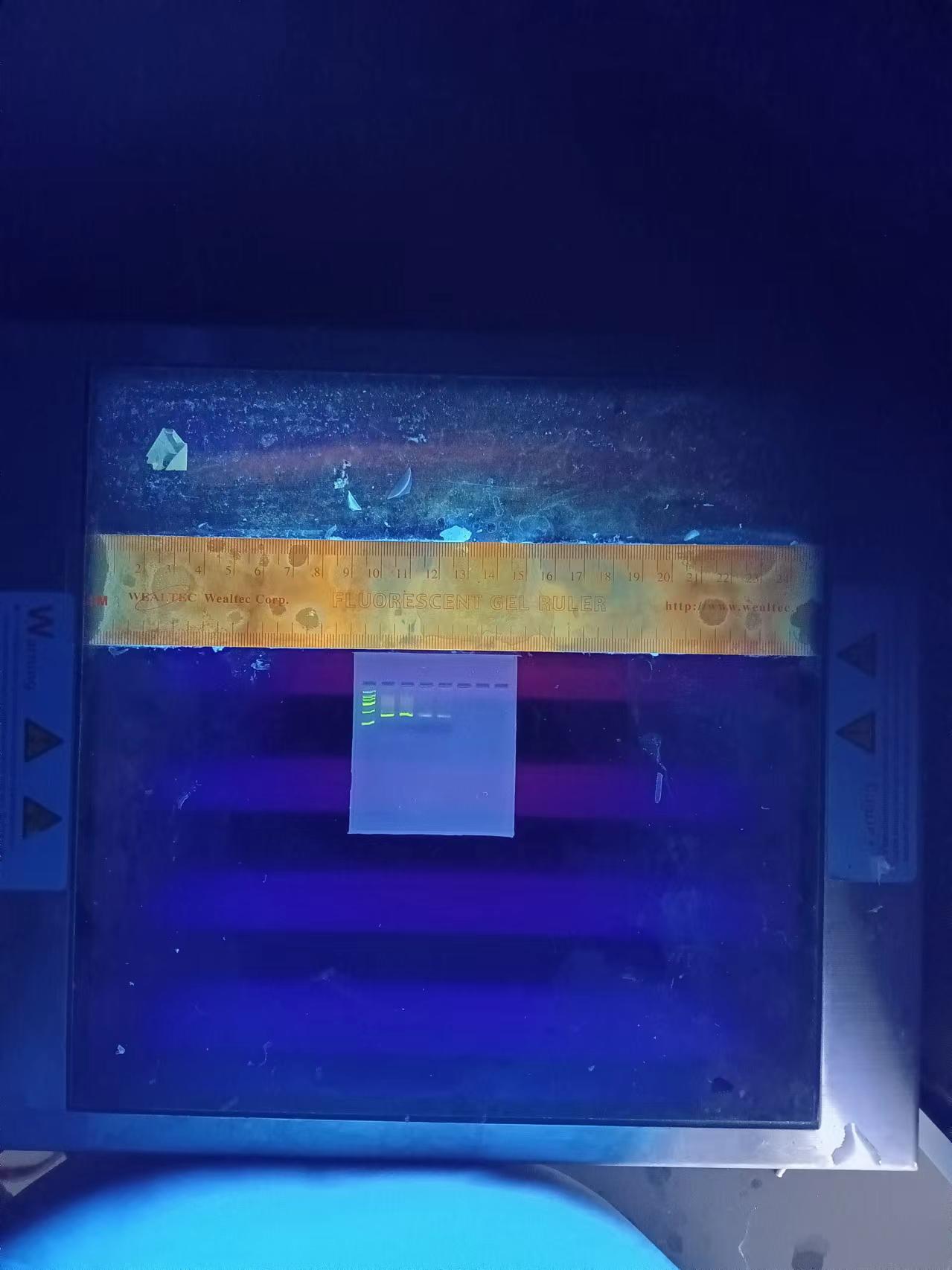


**Figure 2B (uncut) Figure 5B (uncut)**
